# Assessing organisational and technological readiness for artificial intelligence implementation in the Ghana health service: a systematic review protocol

**DOI:** 10.3389/fdgth.2026.1834800

**Published:** 2026-06-15

**Authors:** Victor Luckyboy Dzramado, Obed Uwumbornyi Lasim, Daniel Owusu-Aboagye

**Affiliations:** 1 Department of Biostatistics, Cape Coast Teaching Hospital, Cape Coast, Ghana; 2 Department of Health Services, School of Medical Sciences, University of Cape Coast, Cape Coast, Ghana; 3 Department of Community Health, Kwame Nkrumah University of Science and Technology, Kumasi, Ghana

**Keywords:** artificial intelligence, digital health, eHealth, Ghana health service, health informatics, implementation science, low-and middle-income countries, organisational readiness

## Abstract

**Background:**

Artificial intelligence (AI) holds transformative potential for public health systems in low- and middle-income countries. The Ghana Health Service (GHS), Ghana's principal public health implementing agency, faces persistent workforce capacity gaps, fragmented digital infrastructure, and nascent data governance frameworks that collectively constrain AI adoption. No systematic review has specifically examined the multi-dimensional organisational and technological readiness of the GHS for AI implementation.

**Objectives:**

This systematic review will synthesise evidence on organisational and technological readiness for AI implementation within the GHS and comparable low- and middle-income country contexts, identify barriers and facilitators, document existing AI applications and implementation outcomes, and generate an evidence base to inform national health policy.

**Methods and analysis:**

A systematic review will be conducted following PRISMA 2020 guidelines with protocol registration in PROSPERO (CRD420261339477). A Population-Concept-Context (PCC) framework guides eligibility criteria. Searches were conducted across 15 bibliographic and supplementary sources. Two independent reviewers will conduct double-blind screening with inter-rater reliability assessed using Cohen's kappa, targeting a threshold of 0.80 or above as recommended for complex mixed-methods reviews. Quality appraisal will use validated design-specific tools. A convergent integrated mixed-methods synthesis incorporating thematic synthesis and structured narrative synthesis will be applied, with meta-analysis conducted where sufficient quantitative evidence permits. Certainty of evidence will be assessed using GRADE and GRADE-CERQual.

**Ethics and dissemination:**

Formal ethical approval is not required as this review analyses existing published evidence. Findings will be submitted for peer-reviewed publication and disseminated to the GHS, Ghana Ministry of Health, and WHO Ghana Country Office.

**PROSPERO Registration:**

https://www.crd.york.ac.uk/PROSPERO/view/CRD420261339477, identifier CRD420261339477.

## 1 Introduction

### 1.1 Background and rationale

Artificial intelligence (AI) has emerged as a defining technological development in global health, encompassing machine learning, deep learning, natural language processing, and clinical decision support systems, all of which demonstrate considerable promise across diagnostic, surveillance, resource allocation, and administrative health domains (1). In high-income country settings, AI applications have demonstrated clinical impact in radiology, oncology, and cardiology. At the global health level, the World Health Organization has consistently articulated AI as a mechanism for accelerating universal health coverage, particularly in health systems characterised by severe physician-to-patient disparities and resource constraints (2).

In low- and middle-income countries (LMICs), the potential of AI for health system transformation is simultaneously most urgent and most structurally constrained. Sub-Saharan Africa carries a disproportionate burden of communicable and non-communicable diseases against health systems marked by chronic workforce shortages, inadequate broadband connectivity, fragmented digital infrastructure, and nascent health data governance frameworks (3). These compounding structural realities create a recognised paradox: while AI could theoretically address many of the efficiency and capacity gaps that define LMIC health systems, the same deficits that AI might address are precisely the conditions currently preventing meaningful adoption (4).

The Ghana Health Service (GHS), established under the Ghana Health Service and Teaching Hospitals Act of 1996 (Act 525), is the principal implementing agency of Ghana's national public health system, operating across all 16 administrative regions and managing more than 4,000 health facilities, as documented in official GHS infrastructure records (5). Ghana has made important advances in health digitalisation, including the deployment of the District Health Information Management System (DHIMS2), a National eHealth Policy, and AI-optimised drone supply chain logistics through Zipline in collaboration with the GHS. Despite these advances, the translation of isolated digital health pilots into systematic, scalable AI implementation within GHS operations remains nascent and poorly characterised in the peer-reviewed literature (6).

The concept of readiness for health technology implementation is multi-dimensional. It encompasses organisational dimensions including leadership commitment, institutional culture, change management capacity, and financial resource availability; technological dimensions involving digital infrastructure maturity, hardware access, connectivity, and data quality; and workforce dimensions including AI literacy, technical competency, training availability, and attitudinal acceptance (7). Established implementation science frameworks including the Technology-Organisation-Environment (TOE) Framework (8), the Technology Acceptance Model (TAM) (9), and the Non-adoption, Abandonment, Scale-up, Spread, and Sustainability (NASSS) Framework (10) provide theoretical lenses through which these readiness dimensions can be organised and assessed.

### 1.2 Justification and distinction from related reviews

A search of PROSPERO conducted on 12 March 2026 identified three registered reviews with partial thematic overlap. Each differs substantively from the present protocol and collectively they confirm the gap this review addresses.

The first, a review on AI-enabled epidemic intelligence systems (CRD420251178763), is restricted to epidemic intelligence applications and does not examine organisational or technological readiness dimensions, nor is it situated within a specific national public health institution. Its population focus is on surveillance systems rather than the health workforce and health system infrastructure that form the core of the present review.

The second, a review on AI-driven digital health interventions in low-resource public health systems (CRD42024621423), examines intervention effectiveness rather than readiness assessment. It applies a PICO framework oriented towards evaluating outcomes of deployed interventions, and does not generate evidence on the pre-implementation readiness conditions that must be assessed before deployment decisions are made. The geographic scope is generic across low-resource settings without institutional specificity.

The third, a review on implementation science for responsible AI adoption in healthcare (CRD420250604506), addresses ethical governance and responsible adoption frameworks globally, with no focus on organisational or technological readiness measurement within sub-Saharan African public health system contexts, and no country-specific or institutional analysis.

The present review is uniquely distinguished by four characteristics that, in combination, are not shared by any existing or registered review. First, it applies a multi-dimensional readiness framework rather than an intervention-effectiveness or governance lens. Second, it situates the inquiry within the specific institutional architecture of the GHS, enabling the generation of findings applicable to national policy and operational planning. Third, it incorporates both organisational and technological dimensions within a unified synthesis rather than addressing either domain in isolation. Fourth, it is designed to produce recommendations directly applicable to the GHS strategic planning process and Ghana's emerging national AI healthcare governance framework. The current review is therefore confirmed as warranting independent registration and execution.

### 1.3 Review objectives

This systematic review addresses five pre-specified objectives:

- Assess the current state of organisational readiness of the GHS for AI implementation, including leadership commitment, institutional policies, and change management capacity.
- Evaluate the technological infrastructure and digital readiness of the GHS to support AI adoption, including connectivity, health information systems, and data quality.
- Identify barriers and facilitators to AI implementation within the GHS across workforce, governance, financial, and infrastructural dimensions.
- Synthesise evidence on existing AI applications and pilot projects within the GHS and their implementation outcomes.
- Provide evidence-informed recommendations to strengthen GHS readiness for scalable and sustainable AI implementation aligned with Ghana's national health and digital development agenda.

### 1.4 Review questions

**Primary review question:** What is the current state of organisational and technological readiness of the Ghana Health Service for artificial intelligence implementation?

#### 1.4.1 Secondary review questions

What organisational factors including leadership, policy, governance, and financial resources influence AI readiness within the GHS? What technological and infrastructural factors including connectivity, hardware, health information systems, and data quality characterise the GHS's readiness for AI adoption? What are the key barriers and facilitators to AI implementation within the GHS and comparable Sub-Saharan African public health systems? What AI or AI-enabled digital health applications have been deployed or piloted within the GHS, and what are their documented implementation outcomes? What recommendations emerge from the evidence to strengthen AI readiness within the GHS?

## 2 Methods and analysis

### 2.1 Study design

This study is a systematic review designed and reported in accordance with the Preferred Reporting Items for Systematic Reviews and Meta-Analyses (PRISMA 2020) statement (11). The PRISMA 2020 explanation and elaboration document provides methodological guidance for each reporting item (12). The study follows a convergent integrated mixed-methods synthesis design to accommodate the anticipated heterogeneity of included study designs. The PRISMA-P 2015 checklist guided protocol preparation (13), and the PRISMA-S extension guided the reporting of literature searches (14). The review protocol has been registered with PROSPERO (registration number CRD420261339477; registration date 13 March 2026; full protocol available at https://www.crd.york.ac.uk/PROSPERO/view/CRD420261339477).

Any substantive amendments to the protocol following PROSPERO registration will be documented with version numbers, dates, and rationale, and will be transparently reported in the final systematic review manuscript.

### 2.2 Eligibility criteria

Eligibility criteria are defined using the Population-Concept-Context (PCC) framework, appropriate for reviews examining complex health systems phenomena rather than discrete clinical interventions (15). This framework was selected in preference to the PICO format because the review examines implementation context and readiness dimensions rather than testing the efficacy of a specific intervention against a control.

#### 2.2.1 Population

Studies will include healthcare workers, administrators, policymakers, and information technology professionals employed by or affiliated with the GHS at primary, secondary, or tertiary care levels; GHS health facilities and institutions; studies conducted in Ghana or Sub-Saharan African and LMIC settings with direct transferable relevance to the GHS context; studies reporting on readiness, adoption, implementation, capacity, or use of AI or digital health technologies within public health systems; and studies published from 1 January 2000 to 28 February 2026.

Studies will be excluded if they focus exclusively on private sector health facilities without GHS applicability; address AI implementation in high-income countries without LMIC transferability; involve non-health sectors including agriculture, finance, or education; are editorials, opinion pieces, letters, or commentaries without original data; or were published before 1 January 2000.

Studies with fewer than 10 participants will be excluded. This threshold is consistent with standard practice in systematic reviews of health system readiness, where samples below this size are considered insufficient to establish meaningful patterns of organisational behaviour, inadequately represent the diversity of health workforce perspectives, and carry unacceptable risks of idiosyncratic finding that cannot be transferred to policy planning (16). The threshold is pre-specified to prevent selective application during screening.

#### 2.2.2 Concept

The exposures of interest are the application, deployment, planning, or evaluation of any AI or digital health technology within the GHS or comparable Sub-Saharan African public health systems. This includes clinical decision support systems, AI-assisted diagnostics, predictive modelling for disease surveillance, natural language processing for health records, machine learning for resource allocation, electronic health record (EHR) systems, health information systems, telemedicine and teleconsultation platforms, and AI-enabled mobile or eHealth platforms. Readiness assessments, feasibility studies, needs assessments, and implementation evaluations related to these technologies are included.

Because AI-specific primary research within the GHS is sparse, this review will operationalise AI readiness through proxy indicators drawn from eHealth and EHR implementation studies conducted within the GHS and comparable LMIC health systems (25–30). Evidence on workforce readiness for EHR adoption, digital infrastructure maturity, connectivity sufficiency, leadership attitudes towards digital systems, data quality in existing health information systems, and institutional policy environments for digital technology provides valid and established proxies for the organisational and technological preconditions required for AI deployment, as these foundational conditions necessarily precede and enable AI implementation (4, 7). Studies addressing eHealth or EHR readiness will therefore be included where they report dimensions directly transferable to AI readiness assessment, and all such transferability assessments will be explicitly documented and critically evaluated in the final synthesis. Purely computational or engineering AI algorithm development papers without implementation context, clinical application, or health system analysis will be excluded.

#### 2.2.3 Context

The primary context is the GHS as the principal public health implementing institution in Ghana, operating across all 16 administrative regions. Where direct GHS evidence is limited, studies from comparable Sub-Saharan African or LMIC public health system contexts with demonstrably transferable findings will be considered. The review period spans 1 January 2000 to 28 February 2026, capturing the contemporary digital health and AI era in Ghana.

#### 2.2.4 Eligible study designs

Studies eligible for inclusion are cross-sectional studies, qualitative studies (interviews, focus groups, and ethnographies), mixed-methods studies, case studies, pilot and feasibility studies, cohort studies, programme evaluations, needs assessments, readiness assessments, implementation science studies, and grey literature reports from government and international organisations. Where duplicate publications report the same dataset, only the most methodologically complete report will be retained.

No language restrictions will be applied at the search stage. Non-English language studies identified will be assessed for eligibility using translation services as needed.

### 2.3 Information sources

Searches were conducted across 15 bibliographic and supplementary sources to ensure comprehensive coverage of both peer-reviewed and grey literature (Table 1). All sources were searched individually using database-specific features and controlled vocabulary where available, supplemented by free-text searching. No combined cross-database search strings were used. Each database received a tailored strategy appropriate to its interface.

**Table 1:** Information sources, interfaces, and search dates.

Source	Interface/URL	Coverage	Search date
PubMed/MEDLINE	pubmed.ncbi.nlm.nih.gov	1946 to present	June 2025
CINAHL	ebscohost.com	1937 to present	June 2025
Embase.com	embase.com	1947 to present	June 2025
LILACS	bvsalud.org	1982 to present	June 2025
African Index Medicus (AIM)	aims.who.int/AIM	1993 to present	June 2025
African Journals Online (AJOL)	ajol.info	1997 to present	June 2025
Google Scholar (Standard)	scholar.google.com	Multidisciplinary	June-July 2025
Google Scholar (Comprehensive)	scholar.google.com/advanced	Multidisciplinary	June-July 2025
CABI Digital Library/SearchRxiv	cabidigitallibrary.org	Multidisciplinary	June 2025
WHO IRIS	iris.who.int	Institutional	June 2025
ProQuest Dissertations and Theses	proquest.com	1743 to present	June 2025
Grey literature (GHS/MoH/WHO/World Bank)	Multiple	Varied	July 2025
Reference list checking	Manual (backward citation)	N/A	July 2025
Forward citation tracking	Google Scholar	N/A	July 2025
Journal hand-searching (Ghana Medical Journal; Journal of Public Health in Africa; African Health Sciences)	Manual (TOC browse)	January 2015 to present	July 2025

Scopus and Web of Science were not included in the primary search strategy. This decision reflects the access constraints of the research team operating within the Cape Coast Teaching Hospital and University of Cape Coast institutional framework, where confirmed subscription access during the search period was not available to either database. The African Index Medicus and African Journals Online were prioritised as established repositories of African and Ghanaian health research not consistently indexed in Scopus or Web of Science, thereby addressing the well-documented tendency of health informatics systematic reviews to under-represent African-sourced evidence (17). The sensitivity implications of this decision are explicitly acknowledged as a limitation and are discussed in Section 3.

Subscription limitations may restrict full-text access to Embase and CINAHL at the free-access level; institutional library access at Cape Coast Teaching Hospital and the University of Cape Coast was used to address this. All search strategies were peer reviewed by an independent health information specialist using the PRESS 2015 Checklist before execution (18).

### 2.4 Search strategy

All search strategies were developed in accordance with PRISMA 2020 Item 7 (11) and the PRISMA-S extension (14). A structured three-pillar Boolean search was developed using the PCC framework. Each pillar was constructed using the OR operator to maximise sensitivity within the pillar, and the three pillars are combined using the AND operator to generate the final search. MeSH terms are applied in PubMed/MEDLINE and supplemented by free-text title and abstract searching. Truncation (*) is used to capture term variations. Google Scholar's limitations with complex Boolean syntax are acknowledged and simplified but comprehensive queries were used, with results screened through the first 10 pages per query.

The full PubMed/MEDLINE consolidated search strategy is presented below. Complete strategies for all databases are available in Supplementary File S1 and are embedded in the full protocol at the PROSPERO record.

#### 2.4.1 Pillar 1: AI and digital health technology terms

(“Artificial Intelligence"[MeSH Terms] OR “Machine Learning"[MeSH Terms] OR “Deep Learning"[MeSH Terms] OR “Natural Language Processing"[MeSH Terms] OR “Decision Support Systems, Clinical"[MeSH Terms] OR “Neural Networks, Computer"[MeSH Terms] OR “Diagnosis, Computer-Assisted"[MeSH Terms] OR “Medical Informatics"[MeSH Terms] OR “Telemedicine"[MeSH Terms] OR “Health Information Technology"[MeSH Terms] OR “artificial intelligen*"[Title/Abstract] OR “machine learn*"[Title/Abstract] OR “deep learn*"[Title/Abstract] OR “natural language process*"[Title/Abstract] OR “clinical decision support"[Title/Abstract] OR “computer vision"[Title/Abstract] OR “predictive analytic*"[Title/Abstract] OR “neural network*"[Title/Abstract] OR “digital health technolog*"[Title/Abstract] OR “health informatic*"[Title/Abstract] OR “eHealth"[Title/Abstract] OR “mHealth"[Title/Abstract] OR “AI-enabled"[Title/Abstract] OR “AI-assisted"[Title/Abstract] OR “telehealth"[Title/Abstract] OR “electronic health record*"[Title/Abstract] OR “health information system*"[Title/Abstract]).

The scope of Pillar 1 reflects the deliberate operationalisation of AI readiness through proxy indicators derived from eHealth and EHR readiness, as described in Section 2.2.2. Because the GHS has limited AI-specific primary literature, the inclusion of eHealth and EHR terms in this pillar enables retrieval of the foundational infrastructure and workforce readiness evidence from which AI readiness can be rigorously inferred. All included studies retrieved through these proxy terms will be assessed for transferability to AI readiness as part of the full-text eligibility assessment.

#### 2.4.2 Pillar 2: readiness, implementation, and adoption terms

(“organisational readiness"[Title/Abstract] OR “organizational readiness"[Title/Abstract] OR “technology readiness"[Title/Abstract] OR “digital readiness"[Title/Abstract] OR “implementation readiness"[Title/Abstract] OR “health system readiness"[Title/Abstract] OR “readiness assessment"[Title/Abstract] OR “readiness for change"[Title/Abstract] OR “technology adoption"[Title/Abstract] OR “technology acceptance"[Title/Abstract] OR “Technology Acceptance Model"[Title/Abstract] OR “implementation science"[Title/Abstract] OR “AI adoption"[Title/Abstract] OR “AI implementation"[Title/Abstract] OR “health technology adoption"[Title/Abstract] OR “infrastructure capacity"[Title/Abstract] OR “health workforce capacity"[Title/Abstract] OR “organisational capacity"[Title/Abstract] OR “barriers"[Title/Abstract] AND “facilitators"[Title/Abstract] AND “implement*"[Title/Abstract] OR “digital infrastructure"[Title/Abstract] OR “ICT infrastructure"[Title/Abstract] OR “data governance"[Title/Abstract] AND “health*"[Title/Abstract] OR “AI policy"[Title/Abstract] OR “digital health policy"[Title/Abstract] OR “NASSS framework"[Title/Abstract] OR “TOE framework"[Title/Abstract] AND “health*"[Title/Abstract]).

#### 2.4.3 Pillar 3: geographic and population terms

(“Ghana"[MeSH Terms] OR “Ghana"[Title/Abstract] OR “Ghana Health Service"[Title/Abstract] OR “GHS"[Title/Abstract] AND “Ghana"[Title/Abstract] OR “West Africa"[Title/Abstract] OR “Sub-Saharan Africa"[MeSH Terms] OR “Sub-Saharan Africa"[Title/Abstract] OR “low- and middle-income countr*"[Title/Abstract] OR “LMIC"[Title/Abstract] OR “developing countr*"[Title/Abstract] OR “resource-limited setting*"[Title/Abstract] OR “public health system*"[Title/Abstract] OR “primary health care"[MeSH Terms] OR “health system*"[Title/Abstract] AND “Ghana"[Title/Abstract] OR “Accra"[Title/Abstract] OR “Kumasi"[Title/Abstract]).

#### 2.4.4 Final search: pillar 1 and pillar 2 and pillar 3

The PubMed strategy was validated before execution by confirming retrieval of five pre-identified key papers on AI and digital health in Ghanaian and African health systems (18).

### 2.5 Study selection process

All records retrieved from database searches were exported to Mendeley Reference Manager for deduplication. Automated deduplication was followed by manual verification of potential duplicates. The deduplicated records were screened using a blinded two-stage process.

#### 2.5.1 Stage 1: title and abstract screening

Two independent reviewers (VLD and DO) screened all records at title and abstract level. Each record was classified as Include, Maybe, or Exclude. All records classified as Include or Maybe by either reviewer advanced to full-text assessment. A pilot calibration exercise of 50 title and abstract records was conducted by all three reviewers before commencement of full screening to calibrate eligibility criteria interpretation.

#### 2.5.2 Stage 2: full-text eligibility assessment

Full texts of all records advancing from Stage 1 were independently retrieved and assessed by the same two reviewers against the complete PCC eligibility criteria. Reasons for exclusion at full-text stage were documented for each excluded record and are reported in the PRISMA 2020 flow diagram. Where full texts could not be retrieved despite two contact attempts to corresponding authors and institutional library searches, records were documented as not retrievable.

Disagreements at both stages were resolved through structured discussion between the two primary reviewers. In cases where structured discussion did not achieve consensus, the third reviewer (Prof. OL) provided adjudication as pre-specified in the PROSPERO registration. This three-reviewer arrangement provides a structured and pre-specified adjudication mechanism rather than an additional source of variability; the third reviewer's role is explicitly confined to adjudication of irresolvable disagreements and does not constitute a parallel independent screening track.

Inter-rater reliability was quantified using Cohen's kappa statistic (19, 40). A kappa value at or above 0.80 will be considered indicative of strong agreement and acceptable for proceeding with full screening, consistent with recommended benchmarks for complex mixed-methods screening exercises in systematic reviews (20, 40). This threshold is higher than the Landis and Koch (21) conventional benchmark of 0.61, which has been critiqued as insufficiently demanding for methodologically complex reviews. Where kappa falls below 0.80, a further calibration exercise will be conducted before full screening resumes, and any recalibration will be documented and reported.

**Figure 1:**
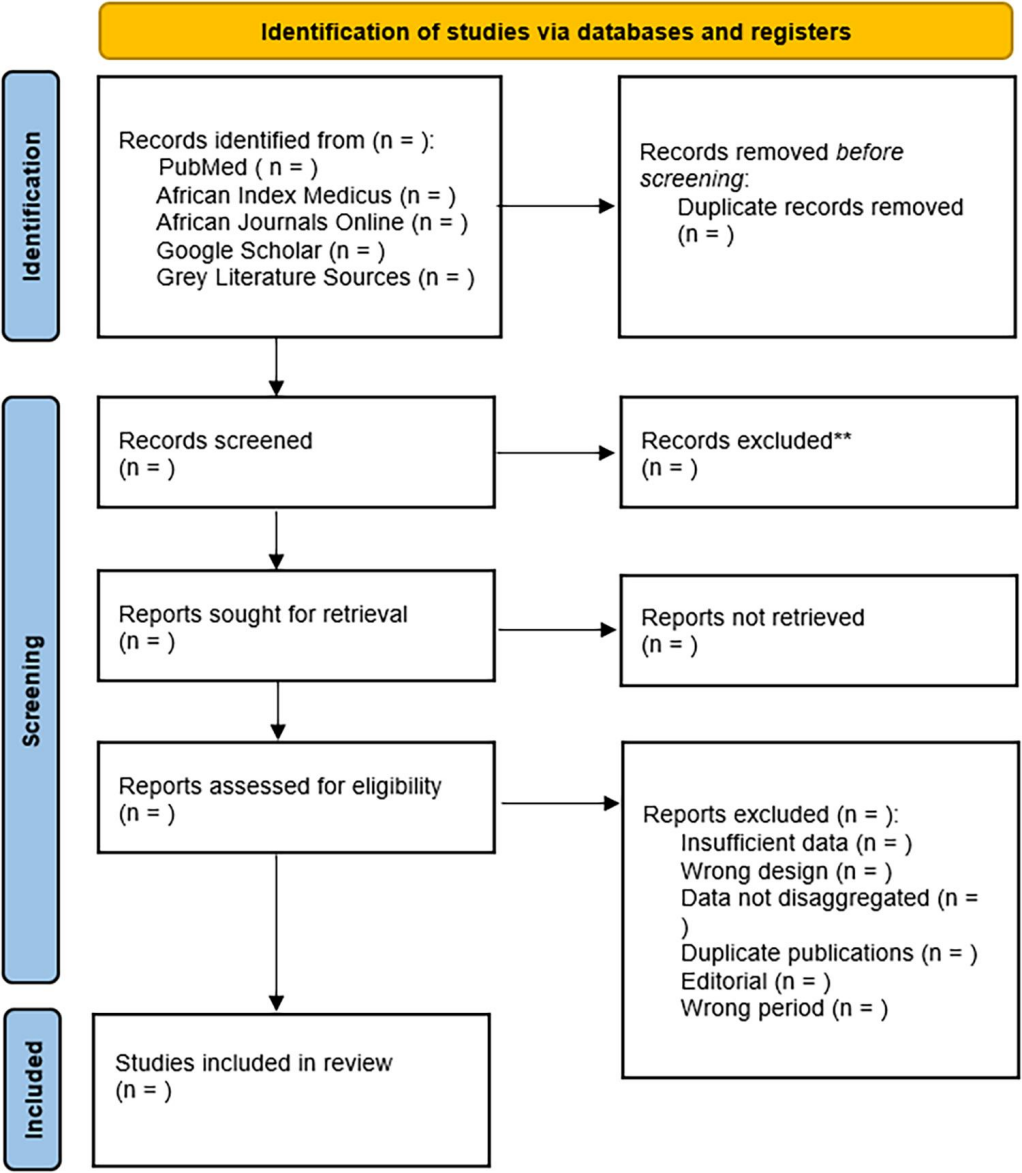
PRISMA 2020 flow diagram.

Figure 1 presents the PRISMA 2020 flow diagram that will be completed on completion of the review. The diagram has been structured to reflect all 15 information sources listed in Table 1, including grey literature and supplementary sources, consistent with PRISMA 2020 recommendations for comprehensive source documentation (11).

### 2.6 Data collection process

A standardised data extraction form was developed in Microsoft Excel and piloted on five included studies before commencement of full extraction. As pre-registered in PROSPERO, data were extracted by one reviewer (VLD) and independently checked by a second reviewer (DO). The form captures the following domains:

Administrative data including study ID, author, year, journal, country, and setting; study characteristics including design, population, sample size, sampling approach, and analytical method; AI or technology details including type of technology, stage of implementation, and application domain; readiness data including framework applied, dimensions assessed, scores, and themes; barriers and facilitators categorised by domain; key outcomes and conclusions; and quality appraisal ratings.

Where data are missing or ambiguous, study authors were contacted by email on up to two occasions over a four-week period, consistent with PROSPERO registration.

### 2.7 Quality appraisal

All included studies were independently assessed for methodological quality by two reviewers (VLD and DO) using validated tools selected according to study design. The use of five appraisal tools reflects the expected methodological heterogeneity of the included studies rather than an arbitrary expansion of the appraisal process: applying a single tool across incompatible study designs would produce unreliable appraisal results and misclassify methodological strengths and weaknesses that are design-specific. The design-specific approach is therefore not a source of methodological inconsistency but rather its prevention (22). Specifically, the Newcastle-Ottawa Scale is applied only to cross-sectional and cohort study designs as intended by its developers; the CASP Qualitative Checklist is applied only to qualitative studies; the MMAT is applied only to mixed-methods studies; ROBIS is applied only to any included systematic or scoping reviews used as background evidence; and the AACODS checklist is applied only to grey literature and descriptive reports. No study type is appraised by more than one instrument (38).

Disagreements were resolved by discussion or adjudication by Prof. OL. Quality appraisal findings are not used to exclude studies but inform sensitivity analyses and certainty of evidence assessment. Table 2 presents the quality appraisal tools assigned by study design.

**Table 2:** Quality appraisal tools by study design.

Study design	Appraisal tool	Key domains assessed
Cross-sectional and cohort studies	Newcastle-Ottawa Scale (adapted for cross-sectional)	Representativeness, measurement, outcome assessment
Qualitative studies	CASP Qualitative Checklist	Rigour, credibility, reflexivity, transferability
Mixed-methods studies	Mixed Methods Appraisal Tool (MMAT Version 2018)	Quantitative component, qualitative component, integration
Systematic and scoping reviews (background evidence)	ROBIS (Risk of Bias in Systematic Reviews)	Scope, methods, reliability, conclusions
Grey literature and descriptive reports	AACODS Checklist	Authority, accuracy, coverage, objectivity, date, significance

### 2.8 Data synthesis

A convergent integrated mixed-methods synthesis will be employed to accommodate the anticipated heterogeneity of study designs, consistent with the strategy pre-registered in PROSPERO. The synthesis proceeds across sequential steps that together constitute a single integrated methodological approach rather than four independent or parallel synthesis strategies. Thematic synthesis and structured narrative synthesis are applied to qualitative and quantitative evidence respectively and are standard components of any mixed-methods synthesis framework (23). Meta-analysis is applied conditionally, only where sufficient quantitative evidence with comparable outcome measures exists, and is not pursued where this condition is not met. Convergent integration is the overarching step that unifies the outputs of the preceding steps into a joint interpretive display. These four steps are therefore methodologically sequential and mutually dependent, not independently competing approaches.

Step 1 applies thematic synthesis to qualitative and mixed-methods study findings, using the approach described by Thomas and Harden (23). This involves line-by-line coding of study findings, development of descriptive themes, and generation of analytical themes that extend interpretively beyond primary study content to produce new constructs about AI readiness in the GHS.

Step 2 applies structured narrative synthesis to quantitative findings where statistical pooling is not feasible, following the EPPI-Centre framework for narrative synthesis (24). Textual description, vote counting, and structured evidence tables are used to compare findings across studies.

Step 3 applies a random-effects meta-analysis only if three or more quantitative studies report comparable outcome measures using validated instruments. Meta-analysis will be conducted using Review Manager (RevMan 5.4) or the R statistical environment using the meta package. Statistical heterogeneity will be quantified using the I2 statistic, with values above 50% indicating moderate heterogeneity and above 75% indicating substantial heterogeneity. Where substantial heterogeneity is identified, meta-analysis will not be reported as a primary finding and the narrative synthesis from Step 2 will be the primary vehicle for quantitative evidence.

Step 4 integrates quantitative and qualitative findings using a convergent synthesis design, producing a joint display matrix mapping quantitative outcomes against qualitative themes to generate comprehensive, policy-relevant conclusions about AI readiness in the GHS.

The NASSS Framework (10) and the Technology-Organisation-Environment (TOE) Framework (8) provide the primary theoretical organising lenses for categorising and interpreting barriers, facilitators, and readiness dimensions identified across included studies. Table 3 presents the pre-specified primary and secondary outcomes.

**Table 3:** Pre-specified primary and secondary outcomes (as registered in PROSPERO CRD420261339477).

Outcome category	Outcome	Measurement approach
Primary	Level of organisational readiness of the GHS for AI implementation	Validated readiness frameworks (WHO-ASSESS, TOE, NASSS); readiness scores, themes, or categories
Primary	Level of technological infrastructure readiness	Digital connectivity, HIS maturity, EHR availability, device access, data quality metrics
Primary	Barriers and facilitators to AI implementation in the GHS	Categorised across organisational, technological, financial, workforce, and policy domains
Primary	Workforce competency and AI literacy among GHS professionals	Surveys, competency assessments, qualitative inquiry
Primary	Existing AI applications deployed within the GHS or comparable facilities	Implementation outcomes and scalability evidence
Secondary	Health policy and governance frameworks for AI in the GHS	National digital health strategies, AI-specific policies, regulatory environments
Secondary	Stakeholder attitudes and perceptions toward AI implementation	Qualitative themes from GHS leadership, clinicians, patients, and community members
Secondary	Financial and resource readiness	Funding mechanisms, budget allocations, public-private partnership models
Secondary	Data governance and ethics readiness	Data protection frameworks, informed consent practices, algorithmic accountability mechanisms
Secondary	Implementation models and frameworks for AI scale-up	Frameworks recommended or tested for the GHS or comparable SSA health systems

### 2.9 Reporting bias assessment

Reporting bias will be assessed in accordance with PRISMA 2020 Item 14 (11). For quantitative studies where meta-analysis is feasible, funnel plot asymmetry will be examined using Egger's test where 10 or more primary studies contribute to a pooled estimate. For qualitative and mixed-methods studies, selective reporting will be assessed narratively by comparing reported findings against stated study objectives. The comprehensiveness of the grey literature search, including searches of GHS and Ghana Ministry of Health websites, WHO, World Bank, and USAID repositories, will be discussed as a contextual mitigation strategy for publication bias.

### 2.10 Certainty of evidence assessment

Certainty of evidence will be assessed in accordance with PRISMA 2020 Item 15 (11). The GRADE approach will be applied to quantitative and mixed-methods evidence. GRADE-CERQual will be applied to qualitative evidence. Two independent reviewers will rate certainty across four domains: risk of bias, coherence, adequacy of data, and relevance. Certainty levels (high, moderate, low, and very low) will be explicitly reported for each major review outcome and presented in a Summary of Findings table. Disagreements in certainty ratings will be resolved by consensus or third-party adjudication.

### 2.11 Pre-specified subgroup and sensitivity analyses

Table 4 presents the pre-specified subgroup and sensitivity analyses for the review.

**Table 4:** Pre-specified subgroup and sensitivity analyses.

Analysis type	Variable	Rationale
Subgroup	Facility level (primary/secondary/tertiary)	Readiness may differ significantly by facility tier within the GHS
Subgroup	Geographic region in Ghana (urban/rural; Greater Accra vs. Northern regions)	Known infrastructure disparities between GHS urban and rural contexts
Subgroup	AI application domain (diagnostics/surveillance/administration/logistics)	Different domains may face distinct readiness barriers
Subgroup	Study design (quantitative/qualitative/mixed-methods)	Methodological source of variation in findings
Sensitivity	Exclude high risk of bias studies	Assess robustness of pooled estimates to study quality
Sensitivity	Restrict to Ghana-only studies	Examine findings from studies exclusively conducted in Ghana
Sensitivity	Restrict to studies published from 2015 onwards	Assess contemporary relevance excluding early pre-digital health era studies

### 2.12 Review timeline and search execution: transparent acknowledgment

The review is registered in PROSPERO with a start date of 1 May 2025 and an end date of 30 April 2026. Formal database searches were conducted between June and August 2025, with grey literature searches and supplementary source searches completed by July 2025. The PROSPERO registration was submitted on 13 March 2026, which is approximately nine months after the formal database searches were completed.

The authors acknowledge that this timeline represents a retrospective rather than prospective protocol registration relative to the search execution date, and that this constitutes a limitation of the present protocol. The searches were conducted according to the strategy described in this protocol, which was substantially developed before search execution commenced in June 2025. The PROSPERO registration formalises the pre-existing protocol and provides public transparency. At the time of registration on 13 March 2026, the stages of pilot work, formal searching, and screening against inclusion criteria had been started and completed. Data extraction, risk of bias assessment, and data synthesis were in progress.

To minimise the risk of *post-hoc* adjustment of eligibility criteria or synthesis decisions, the authors confirm that the eligibility criteria, search strategy, and synthesis approach described in this protocol are identical to those applied during the screening and extraction process. The PROSPERO record documents the primary protocol parameters as registered. No amendments to eligibility criteria, search strings, or synthesis methods were made following the initiation of screening. This limitation will be explicitly disclosed in the final systematic review manuscript, consistent with recommended practice for retrospective registration transparency (11, 13).

### 2.13 Patient and public involvement

This systematic review analyses existing published literature and does not involve primary data collection from patients or members of the public. Patient and public involvement in the design or conduct of this review was therefore not applicable.

## 3 Discussion

This protocol outlines the methodology for a systematic review addressing a substantive gap in the public health literature on AI implementation in Ghana. While broader reviews of AI in African healthcare exist and digital health readiness in LMIC settings has received growing scholarly attention, the specific focus on organisational and technological readiness within the institutional context of the Ghana Health Service has not previously been the subject of a systematic synthesis. As documented in Section 1.2, three related PROSPERO-registered reviews differ from this protocol in population focus, intervention scope, outcome specification, and institutional specificity; none addresses the combined organisational and technological readiness dimensions within the GHS. This review addresses a confirmed and specific evidence gap.

Several methodological considerations merit prospective discussion. The anticipated heterogeneity of study designs reflects the evolving and interdisciplinary nature of the literature on digital health readiness in LMIC settings. This heterogeneity is the principal justification for the convergent mixed-methods synthesis approach, which is capable of honouring the nuances of both qualitative and quantitative evidence rather than forcing artificial equivalence between very different types of studies. The theoretical frameworks selected for the synthesis, the NASSS Framework (10) and the TOE Framework (8), provide complementary analytical perspectives: the TOE framework illuminates structural determinants of technology adoption within organisations, while the NASSS framework captures the dynamic, non-linear trajectories of health technology implementation including the frequently under-studied phenomena of non-adoption and abandonment.

The decision to extend the geographic scope to include comparable Sub-Saharan African and LMIC public health systems, where direct GHS evidence is limited, is justified by the recognised scarcity of Ghana-specific AI implementation studies and the need to generate actionable, policy-relevant conclusions (31–36). The transferability of findings from comparable contexts will be explicitly examined and critically reported rather than assumed. The comprehensive multi-database search strategy, incorporating the African Index Medicus and African Journals Online alongside major biomedical databases, is designed to address the well-documented tendency of health informatics systematic reviews to under-represent African-sourced evidence (17).

This review has several prospective limitations. First, the retrospective nature of PROSPERO registration relative to search execution, as detailed in Section 2.12, represents the primary methodological limitation of this protocol. The authors have documented this transparently and confirm that no modifications were made to eligibility criteria or synthesis design following search initiation. Second, the absence of Scopus and Web of Science from the primary search strategy, driven by subscription access constraints, may reduce retrieval sensitivity for grey literature and non-African-indexed studies; this is partially mitigated by the extensive African-specific database coverage and grey literature searches described in Table 1 (37, 39). Third, the nascent state of AI implementation within the GHS means that AI-specific primary research is sparse; the majority of evidence addressing the review questions is drawn from broader digital health or eHealth readiness studies, from which AI readiness is inferred through carefully documented transferability assessment. Workforce readiness estimates will be drawn from eHealth and EHR readiness studies that may not fully capture AI-specific competency requirements, and this limitation will be explicitly characterised when presenting individual study findings (25, 27, 29). Fourth, subscription limitations may constrain access to all records in Embase and CINAHL, though institutional library access mitigated this during search execution.

The GHS manages more than 4,000 health facilities across 16 administrative regions, as documented in the Ghana Health Service Annual Report (5), providing the organisational scale that makes systematic AI readiness assessment both necessary and policy-significant. The anticipated findings of this review will generate direct utility for GHS strategic planning, for Ghana's emerging national AI governance framework, and for international actors investing in digital health systems strengthening in West Africa. By synthesising what is currently known and mapping where evidence is absent, the review will identify priority areas for future primary research on AI readiness in Ghana and comparable LMIC health systems. The review findings will be directly applicable to the Ghana Ministry of Health's forthcoming National AI Healthcare Policy development and to the update of the Ghana Health Sector Medium-Term Development Plan.

## 4 Ethics and dissemination

This systematic review synthesises existing published and publicly available literature and does not involve primary data collection from human participants, animal subjects, or personal health data. Formal ethical approval is therefore not required. All included published materials will be used in accordance with copyright and intellectual property provisions. Authorship attribution will comply with the International Committee of Medical Journal Editors (ICMJE) guidelines for authorship. No conflicts of interest have been declared by any member of the review team, as recorded in the PROSPERO registration.

Findings will be disseminated through the following channels. The primary vehicle will be submission of the completed systematic review for publication in a peer-reviewed journal. The PROSPERO registered protocol (CRD420261339477) is publicly accessible. Key findings will be translated into evidence-informed policy briefs targeted at the Ghana Health Service headquarters, the Ghana Ministry of Health, and the WHO Ghana Country Office. The review team intends to engage relevant international development partners including GIZ, the World Bank, and the Global Fund to Fight AIDS, Tuberculosis and Malaria as planned dissemination recipients; however, no formal engagement or commitment from these organisations has been established at the time of this protocol submission, and these represent intended rather than confirmed dissemination channels. Findings will additionally be presented at the Ghana Public Health Association annual conference and relevant international digital health forums. All co-authors have consented to submission and publication of this protocol manuscript.
