# Supplementary material for: Assessing organisational and technological readiness for artificial intelligence implementation in the Ghana health service: a systematic review protocol
Source: Front Digit Health. 2026 Jun 15;8:1834800. doi: 10.3389/fdgth.2026.1834800 (PMC13311085; doi:10.3389/fdgth.2026.1834800)
Supplement: Supplementary file 1 [file Supplementaryfile1.docx]

**Supplementary File S1: Complete Database Search Strategies**

**Manuscript:** Assessing Organisational and Technological Readiness for Artificial Intelligence Implementation in the Ghana Health Service: A Systematic Review Protocol

**Journal:** Frontiers in Digital Health, Section: Health Informatics

**PROSPERO Registration:** CRD420261339477

**Corresponding Author:** Victor Luckyboy Dzramado (mldzramado@st.knust.edu.gh)

**Date of Search Execution:** June to August 2025 (grey literature: July 2025)

**Reporting Standard:** PRISMA-S 2021 Extension for Reporting Literature Searches in Systematic Reviews (Rethlefsen et al., Syst Rev. 2021;10:39)

All search strategies were developed using a three-pillar Boolean structure based on the Population-Concept-Context (PCC) framework. Each pillar was constructed using the OR operator to maximise sensitivity within the pillar; the three pillars were combined using the AND operator. All strategies were peer reviewed by an independent health information specialist using the PRESS 2015 Checklist prior to execution. The strategies below are presented individually for each of the 15 information sources searched.

**S1.1 PubMed/MEDLINE**

**Interface:** pubmed.ncbi.nlm.nih.gov

**Date searched:** June 2025

**Coverage period searched:** 1 January 2000 to 28 February 2026

**Controlled vocabulary applied:** Medical Subject Headings (MeSH)

**Records retrieved (pre-deduplication):** See PRISMA 2020 flow diagram

**Pillar 1: AI and Digital Health Technology Terms**

("Artificial Intelligence"[MeSH Terms] OR "Machine Learning"[MeSH Terms] OR "Deep Learning"[MeSH Terms] OR "Natural Language Processing"[MeSH Terms] OR "Decision Support Systems, Clinical"[MeSH Terms] OR "Neural Networks, Computer"[MeSH Terms] OR "Diagnosis, Computer-Assisted"[MeSH Terms] OR "Medical Informatics"[MeSH Terms] OR "Telemedicine"[MeSH Terms] OR "Health Information Technology"[MeSH Terms] OR "artificial intelligen*"[Title/Abstract] OR "machine learn*"[Title/Abstract] OR "deep learn*"[Title/Abstract] OR "natural language process*"[Title/Abstract] OR "clinical decision support"[Title/Abstract] OR "computer vision"[Title/Abstract] OR "predictive analytic*"[Title/Abstract] OR "neural network*"[Title/Abstract] OR "digital health technolog*"[Title/Abstract] OR "health informatic*"[Title/Abstract] OR "eHealth"[Title/Abstract] OR "e-health"[Title/Abstract] OR "mHealth"[Title/Abstract] OR "m-health"[Title/Abstract] OR "AI-enabled"[Title/Abstract] OR "AI-assisted"[Title/Abstract] OR "telehealth"[Title/Abstract] OR "telemedicine"[Title/Abstract] OR "electronic health record*"[Title/Abstract] OR "EHR"[Title/Abstract] OR "health information system*"[Title/Abstract])

**Pillar 2: Readiness, Implementation, and Adoption Terms**

("organisational readiness"[Title/Abstract] OR "organizational readiness"[Title/Abstract] OR "technology readiness"[Title/Abstract] OR "digital readiness"[Title/Abstract] OR "implementation readiness"[Title/Abstract] OR "health system readiness"[Title/Abstract] OR "readiness assessment"[Title/Abstract] OR "readiness for change"[Title/Abstract] OR "technology adoption"[Title/Abstract] OR "technology acceptance"[Title/Abstract] OR "Technology Acceptance Model"[Title/Abstract] OR "implementation science"[Title/Abstract] OR "AI adoption"[Title/Abstract] OR "AI implementation"[Title/Abstract] OR "health technology adoption"[Title/Abstract] OR "infrastructure capacity"[Title/Abstract] OR "health workforce capacity"[Title/Abstract] OR "organisational capacity"[Title/Abstract] OR "organizational capacity"[Title/Abstract] OR ("barriers"[Title/Abstract] AND "facilitators"[Title/Abstract] AND "implement*"[Title/Abstract]) OR ("enablers"[Title/Abstract] AND "health technolog*"[Title/Abstract]) OR "digital infrastructure"[Title/Abstract] OR "ICT infrastructure"[Title/Abstract] OR ("data governance"[Title/Abstract] AND "health*"[Title/Abstract]) OR "AI policy"[Title/Abstract] OR "digital health policy"[Title/Abstract] OR "NASSS framework"[Title/Abstract] OR ("TOE framework"[Title/Abstract] AND "health*"[Title/Abstract]))

**Pillar 3: Geographic and Population Terms**

("Ghana"[MeSH Terms] OR "Ghana"[Title/Abstract] OR "Ghana Health Service"[Title/Abstract] OR ("GHS"[Title/Abstract] AND "Ghana"[Title/Abstract]) OR "West Africa"[Title/Abstract] OR "Sub-Saharan Africa"[MeSH Terms] OR "Sub-Saharan Africa"[Title/Abstract] OR "low- and middle-income countr*"[Title/Abstract] OR "LMIC"[Title/Abstract] OR "developing countr*"[Title/Abstract] OR "resource-limited setting*"[Title/Abstract] OR "resource-constrained setting*"[Title/Abstract] OR "public health system*"[Title/Abstract] OR "primary health care"[MeSH Terms] OR ("health system*"[Title/Abstract] AND ("Ghana"[Title/Abstract] OR "Africa"[Title/Abstract])) OR ("community health"[Title/Abstract] AND "Ghana"[Title/Abstract]) OR ("district health"[Title/Abstract] AND "Ghana"[Title/Abstract]) OR "Accra"[Title/Abstract] OR "Kumasi"[Title/Abstract])

**FINAL SEARCH:** Pillar 1 AND Pillar 2 AND Pillar 3

**Date limits applied:** 2000/01/01 to 2026/02/28

**Language limits:** None

**Strategy validation:** Confirmed retrieval of five pre-identified key papers before execution, consistent with PRESS 2015 Checklist guidance.

**S1.2 CINAHL (Cumulative Index to Nursing and Allied Health Literature)**

**Interface:** EBSCOhost (ebscohost.com)

**Date searched:** June 2025

**Coverage period searched:** 1 January 2000 to 28 February 2026

**Controlled vocabulary applied:** CINAHL Subject Headings (CSH)

**Access:** Institutional library access via Cape Coast Teaching Hospital and University of Cape Coast

**Pillar 1: AI and Digital Health Technology Terms**

(MH "Artificial Intelligence" OR MH "Machine Learning" OR MH "Natural Language Processing" OR MH "Decision Support Systems, Clinical" OR MH "Neural Networks (Computer)" OR MH "Medical Informatics" OR MH "Telemedicine" OR MH "Health Information Systems" OR TI "artificial intelligen*" OR AB "artificial intelligen*" OR TI "machine learn*" OR AB "machine learn*" OR TI "deep learn*" OR AB "deep learn*" OR TI "natural language process*" OR AB "natural language process*" OR TI "clinical decision support" OR AB "clinical decision support" OR TI "computer vision" OR AB "computer vision" OR TI "predictive analytic*" OR AB "predictive analytic*" OR TI "neural network*" OR AB "neural network*" OR TI "digital health technolog*" OR AB "digital health technolog*" OR TI "health informatic*" OR AB "health informatic*" OR TI "eHealth" OR AB "eHealth" OR TI "e-health" OR AB "e-health" OR TI "mHealth" OR AB "mHealth" OR TI "m-health" OR AB "m-health" OR TI "AI-enabled" OR AB "AI-enabled" OR TI "AI-assisted" OR AB "AI-assisted" OR TI "telehealth" OR AB "telehealth" OR TI "telemedicine" OR AB "telemedicine" OR TI "electronic health record*" OR AB "electronic health record*" OR TI "EHR" OR AB "EHR" OR TI "health information system*" OR AB "health information system*")

**Pillar 2: Readiness, Implementation, and Adoption Terms**

(TI "organisational readiness" OR AB "organisational readiness" OR TI "organizational readiness" OR AB "organizational readiness" OR TI "technology readiness" OR AB "technology readiness" OR TI "digital readiness" OR AB "digital readiness" OR TI "implementation readiness" OR AB "implementation readiness" OR TI "health system readiness" OR AB "health system readiness" OR TI "readiness assessment" OR AB "readiness assessment" OR TI "technology adoption" OR AB "technology adoption" OR TI "technology acceptance" OR AB "technology acceptance" OR TI "Technology Acceptance Model" OR AB "Technology Acceptance Model" OR TI "implementation science" OR AB "implementation science" OR TI "AI adoption" OR AB "AI adoption" OR TI "AI implementation" OR AB "AI implementation" OR TI "health technology adoption" OR AB "health technology adoption" OR TI "digital infrastructure" OR AB "digital infrastructure" OR TI "ICT infrastructure" OR AB "ICT infrastructure" OR TI "AI policy" OR AB "AI policy" OR TI "digital health policy" OR AB "digital health policy" OR TI "NASSS framework" OR AB "NASSS framework" OR TI "TOE framework" OR AB "TOE framework")

**Pillar 3: Geographic and Population Terms**

(TI "Ghana" OR AB "Ghana" OR TI "Ghana Health Service" OR AB "Ghana Health Service" OR TI "West Africa" OR AB "West Africa" OR TI "Sub-Saharan Africa" OR AB "Sub-Saharan Africa" OR MH "Africa South of the Sahara" OR TI "low- and middle-income countr*" OR AB "low- and middle-income countr*" OR TI "LMIC" OR AB "LMIC" OR TI "developing countr*" OR AB "developing countr*" OR TI "resource-limited setting*" OR AB "resource-limited setting*" OR TI "public health system*" OR AB "public health system*" OR MH "Primary Health Care" OR TI "Accra" OR AB "Accra" OR TI "Kumasi" OR AB "Kumasi")

**FINAL SEARCH:** Pillar 1 AND Pillar 2 AND Pillar 3

**Date limits applied:** 2000 to 2026

**Language limits:** None

**S1.3 Embase**

**Interface:** embase.com

**Date searched:** June 2025

**Coverage period searched:** 1 January 2000 to 28 February 2026

**Controlled vocabulary applied:** Emtree

**Access:** Institutional library access via Cape Coast Teaching Hospital and University of Cape Coast

**Pillar 1: AI and Digital Health Technology Terms**

('artificial intelligence'/exp OR 'machine learning'/exp OR 'deep learning'/exp OR 'natural language processing'/exp OR 'clinical decision support system'/exp OR 'neural network'/exp OR 'medical informatics'/exp OR 'telemedicine'/exp OR 'health information system'/exp OR 'artificial intelligen*':ti,ab OR 'machine learn*':ti,ab OR 'deep learn*':ti,ab OR 'natural language process*':ti,ab OR 'clinical decision support':ti,ab OR 'predictive analytic*':ti,ab OR 'computer vision':ti,ab OR 'neural network*':ti,ab OR 'digital health technolog*':ti,ab OR 'health informatic*':ti,ab OR 'ehealth':ti,ab OR 'e-health':ti,ab OR 'mhealth':ti,ab OR 'm-health':ti,ab OR 'ai-enabled':ti,ab OR 'ai-assisted':ti,ab OR 'telehealth':ti,ab OR 'electronic health record*':ti,ab OR 'ehr':ti,ab OR 'health information system*':ti,ab)

**Pillar 2: Readiness, Implementation, and Adoption Terms**

('organisational readiness':ti,ab OR 'organizational readiness':ti,ab OR 'technology readiness':ti,ab OR 'digital readiness':ti,ab OR 'implementation readiness':ti,ab OR 'health system readiness':ti,ab OR 'readiness assessment':ti,ab OR 'readiness for change':ti,ab OR 'technology adoption':ti,ab OR 'technology acceptance':ti,ab OR 'technology acceptance model':ti,ab OR 'implementation science':ti,ab OR 'ai adoption':ti,ab OR 'ai implementation':ti,ab OR 'health technology adoption':ti,ab OR 'infrastructure capacity':ti,ab OR 'health workforce capacity':ti,ab OR 'organisational capacity':ti,ab OR 'organizational capacity':ti,ab OR 'digital infrastructure':ti,ab OR 'ict infrastructure':ti,ab OR 'data governance':ti,ab AND 'health*':ti,ab OR 'ai policy':ti,ab OR 'digital health policy':ti,ab OR 'nasss framework':ti,ab OR 'toe framework':ti,ab AND 'health*':ti,ab)

**Pillar 3: Geographic and Population Terms**

('ghana'/exp OR 'ghana':ti,ab OR 'ghana health service':ti,ab OR 'west africa':ti,ab OR 'sub-saharan africa'/exp OR 'sub-saharan africa':ti,ab OR 'low and middle income countr*':ti,ab OR 'lmic':ti,ab OR 'developing countr*':ti,ab OR 'resource-limited setting*':ti,ab OR 'resource-constrained setting*':ti,ab OR 'public health system*':ti,ab OR 'primary health care'/exp OR 'accra':ti,ab OR 'kumasi':ti,ab)

**FINAL SEARCH:** Pillar 1 AND Pillar 2 AND Pillar 3

**Date limits applied:** 2000 to 2026

**Language limits:** None

**S1.4 LILACS (Literatura Latino-Americana e do Caribe em Ciencias da Saude)**

**Interface:** Biblioteca Virtual em Saude (bvsalud.org)

**Date searched:** June 2025

**Coverage period searched:** 1 January 2000 to 28 February 2026

**Controlled vocabulary applied:** DeCS (Descriptores en Ciencias de la Salud)

**Pillar 1: AI and Digital Health Technology Terms**

(tw:("artificial intelligence" OR "machine learning" OR "deep learning" OR "natural language processing" OR "clinical decision support" OR "neural network" OR "predictive analytics" OR "computer-aided diagnosis" OR "eHealth" OR "e-health" OR "mHealth" OR "m-health" OR "digital health" OR "health informatics" OR "health information system" OR "telemedicine" OR "telehealth" OR "teleconsultation" OR "electronic health record" OR "EHR" OR "EMR" OR "health technology" OR "digital transformation" OR "intelligent system" OR "inteligencia artificial" OR "aprendizaje automatico" OR "salud digital" OR "informatica medica" OR "telemedicina" OR "registros de salud electronica"))

**Pillar 2: Readiness, Implementation, and Adoption Terms**

(tw:("organisational readiness" OR "organizational readiness" OR "technology readiness" OR "digital readiness" OR "implementation readiness" OR "readiness assessment" OR "technology adoption" OR "technology acceptance" OR "Technology Acceptance Model" OR "implementation science" OR "AI adoption" OR "AI implementation" OR "health technology adoption" OR "infrastructure capacity" OR "health workforce capacity" OR "digital infrastructure" OR "ICT infrastructure" OR "data governance" OR "AI policy" OR "digital health policy" OR "barriers" OR "facilitators" OR "enablers" OR "implementacao" OR "adocao de tecnologia" OR "preparacao organizacional"))

**Pillar 3: Geographic and Population Terms**

(tw:("Ghana" OR "Ghana Health Service" OR "West Africa" OR "Sub-Saharan Africa" OR "Africa Subsaariana" OR "low- and middle-income countr*" OR "LMIC" OR "developing countr*" OR "resource-limited setting*" OR "public health system*" OR "paises de bajos ingresos" OR "sistema de salud publica" OR "Africa Occidental"))

**FINAL SEARCH:** Pillar 1 AND Pillar 2 AND Pillar 3

**Date limits applied:** 2000 to 2026

**Language limits:** None

**S1.5 African Index Medicus (AIM)**

**Interface:** aims.who.int/AIM

**Date searched:** June 2025

**Coverage period searched:** 1 January 2000 to 28 February 2026

**Controlled vocabulary applied:** Free-text searching; AIM does not support MeSH

**Note:** The AIM search strategy was executed as documented below, reflecting the interface-specific free-text search capabilities of the African Index Medicus, which does not support complex Boolean operators in the same manner as PubMed. Multiple searches were executed and results were combined manually. The strategy below represents the combined three-pillar query applied across four separate search executions to maximise coverage.

**Search Query (applied as executed across multiple AIM search sessions):**

Pillar 1:

("artificial intelligence" OR "machine learning" OR "deep learning" OR "natural language processing" OR "clinical decision support" OR "neural network" OR "predictive analytics" OR "computer-aided diagnosis" OR "eHealth" OR "e-health" OR "mHealth" OR "m-health" OR "digital health" OR "health informatics" OR "health information system" OR "telemedicine" OR "telehealth" OR "teleconsultation" OR "electronic health record" OR "EHR" OR "EMR" OR "health technology" OR "digital transformation" OR "intelligent system")

AND

Pillar 2:

("readiness" OR "organizational readiness" OR "organisational readiness" OR "technology readiness" OR "digital readiness" OR "AI readiness" OR "implementation" OR "adoption" OR "integration" OR "deployment" OR "barriers" OR "challenges" OR "facilitators" OR "enablers" OR "drivers" OR "capacity" OR "infrastructure" OR "workforce" OR "human resource" OR "technology acceptance" OR "digital transformation" OR "scale-up" OR "policy" OR "governance" OR "sustainability" OR "feasibility")

AND

Pillar 3:

("Ghana" OR "Ghana Health Service" OR "West Africa" OR "Sub-Saharan Africa" OR "Africa" AND ("health system" OR "public health") OR "low income" OR "middle income" OR "LMIC" OR "developing countries" OR "resource-limited" OR "resource-constrained" OR "primary health care" OR "community health" OR "district health")

**Date limits applied:** 2000 to 2026

**Language limits:** None

**S1.6 African Journals Online (AJOL)**

**Interface:** ajol.info

**Date searched:** June 2025

**Coverage period searched:** 1 January 2000 to 28 February 2026

**Controlled vocabulary applied:** Free-text searching

**Search executed using AJOL advanced search:**

**Search terms applied in title, abstract, and keywords fields:**

("artificial intelligence" OR "machine learning" OR "deep learning" OR "clinical decision support" OR "eHealth" OR "mHealth" OR "digital health" OR "health informatics" OR "health information system" OR "telemedicine" OR "electronic health record" OR "EHR")

AND

("readiness" OR "organizational readiness" OR "technology adoption" OR "technology acceptance" OR "implementation" OR "barriers" OR "facilitators" OR "capacity" OR "digital infrastructure" OR "workforce" OR "AI implementation" OR "AI adoption")

AND

("Ghana" OR "Ghana Health Service" OR "West Africa" OR "Sub-Saharan Africa" OR "low- and middle-income" OR "LMIC" OR "developing countries" OR "resource-limited" OR "primary health care")

**Date limits applied:** 2000 to 2026

**Language limits:** None

**Note:** AJOL search results were supplemented by hand-searching of the Ghana Medical Journal and Journal of Public Health in Africa, documented separately under S1.15.

**S1.7 Google Scholar (Standard Search)**

**Interface:** scholar.google.com

**Date searched:** June to July 2025

**Coverage period searched:** 2000 to 2026

**Note:** Google Scholar does not support full Boolean syntax. Searches were conducted using the standard interface with simplified but comprehensive queries. Results were screened through the first ten pages (100 results) per query, consistent with established systematic review practice for Google Scholar. The following individual queries were executed sequentially and results were combined for deduplication.

**Query 1 (AI readiness, Ghana):**

artificial intelligence readiness Ghana Health Service implementation

**Query 2 (digital health implementation, Ghana):**

digital health eHealth implementation barriers facilitators Ghana

**Query 3 (AI adoption, sub-Saharan Africa):**

AI adoption implementation readiness sub-Saharan Africa health system

**Query 4 (organisational readiness, LMIC):**

organizational readiness artificial intelligence low middle income countries health

**Query 5 (EHR readiness, Ghana):**

electronic health records readiness Ghana health facilities

**Query 6 (health informatics, West Africa):**

health informatics technology adoption West Africa public health

**Query 7 (workforce AI literacy, Africa):**

health workforce AI literacy digital readiness Africa

**Query 8 (telemedicine implementation, Ghana):**

telemedicine teleconsultation implementation Ghana barriers

**Date limits applied:** From 2000; applied using Google Scholar date filter tools

**Language limits:** None

**S1.8 Google Scholar (Comprehensive Advanced Search)**

**Interface:** scholar.google.com/advanced_scholar_search

**Date searched:** June to July 2025

**Coverage period searched:** 2000 to 2026

**Note:** The advanced search interface was used to apply more structured queries combining exact phrases, author field searching, and date range restrictions. Results were screened through the first ten pages per query and combined with S1.7 results for deduplication.

**Advanced Query 1:**

All of the words: readiness implementation artificial intelligence

Exact phrase: Ghana Health Service

Date range: 2000 to 2026

**Advanced Query 2:**

All of the words: barriers facilitators digital health implementation

Exact phrase: Sub-Saharan Africa

Date range: 2000 to 2026

**Advanced Query 3:**

All of the words: technology acceptance model health information system

At least one of the words: Ghana Africa LMIC

Date range: 2000 to 2026

**Advanced Query 4:**

All of the words: organizational readiness AI machine learning health

At least one of the words: Ghana Africa low-income

Date range: 2000 to 2026

**Advanced Query 5:**

All of the words: eHealth mHealth implementation readiness workforce

At least one of the words: Ghana West Africa

Date range: 2000 to 2026

**Date limits applied:** 2000 to 2026

**Language limits:** None

**S1.9 CABI Digital Library / SearchRxiv**

**Interface:** cabidigitallibrary.org

**Date searched:** June 2025

**Coverage period searched:** Multidisciplinary; searched without date restriction then filtered to 2000 to 2026

**Note:** The CABI Digital Library SearchRxiv interface accepts free-text and Boolean queries. The search was submitted via the SearchRxiv submission interface as documented below. The interface does not support MeSH or controlled vocabulary.

**Search Query:**

("artificial intelligence" OR "machine learning" OR "deep learning" OR "eHealth" OR "mHealth" OR "digital health" OR "health information system" OR "electronic health record" OR "telemedicine" OR "clinical decision support")

AND

("readiness" OR "organizational readiness" OR "implementation" OR "adoption" OR "barriers" OR "facilitators" OR "capacity" OR "technology acceptance" OR "digital infrastructure" OR "workforce")

AND

("Ghana" OR "Ghana Health Service" OR "Sub-Saharan Africa" OR "West Africa" OR "low- and middle-income" OR "LMIC" OR "developing countries" OR "Africa")

**Date limits applied:** 2000 to 2026

**Language limits:** None

**S1.10 WHO IRIS (Institutional Repository for Information Sharing)**

**Interface:** iris.who.int

**Date searched:** June 2025

**Coverage period searched:** Institutional coverage searched without restriction, results filtered to 2000 to 2026

**Note:** WHO IRIS supports free-text and field-based searching. Controlled vocabulary is not available. The following queries were executed sequentially.

**Query 1:**

"artificial intelligence" OR "AI" AND "Ghana" AND "health"

**Query 2:**

"digital health" OR "eHealth" AND "Ghana Health Service" OR "Ghana"

**Query 3:**

"AI readiness" OR "digital readiness" OR "health technology adoption" AND "Africa" AND "low-income"

**Query 4:**

"health information system" OR "electronic health record" AND "Ghana" AND "implementation"

**Query 5:**

"technology adoption" OR "organizational readiness" AND "Sub-Saharan Africa" AND "health"

**Collections searched:** WHO technical reports, policy documents, working papers, and regional office publications (AFRO)

**Date limits applied:** 2000 to 2026

**Language limits:** None

**S1.11 ProQuest Dissertations and Theses Global**

**Interface:** proquest.com/pqdtglobal

**Date searched:** June 2025

**Coverage period searched:** 1 January 2000 to 28 February 2026

**Controlled vocabulary applied:** ProQuest subject headings; supplemented by free-text searching

**Pillar 1 (condensed for ProQuest interface):**

ab("artificial intelligence" OR "machine learning" OR "deep learning" OR "clinical decision support" OR "eHealth" OR "mHealth" OR "digital health" OR "health information system" OR "electronic health record" OR "telemedicine" OR "health informatics" OR "AI-enabled")

**Pillar 2 (condensed for ProQuest interface):**

ab("organizational readiness" OR "organisational readiness" OR "technology readiness" OR "digital readiness" OR "implementation readiness" OR "technology adoption" OR "technology acceptance" OR "implementation science" OR "AI implementation" OR "AI adoption" OR "barriers" AND "facilitators" AND "implement*" OR "digital infrastructure" OR "health workforce capacity" OR "AI policy")

**Pillar 3 (condensed for ProQuest interface):**

ab("Ghana" OR "Ghana Health Service" OR "West Africa" OR "Sub-Saharan Africa" OR "LMIC" OR "low- and middle-income" OR "developing countries" OR "resource-limited" OR "primary health care" OR "Accra" OR "Kumasi")

**FINAL SEARCH:** Pillar 1 AND Pillar 2 AND Pillar 3

**Document types searched:** Dissertations; theses; masters theses; doctoral dissertations

**Date limits applied:** 2000 to 2026

**Language limits:** None

**S1.12 Grey Literature Sources**

**Sources searched:** Ghana Health Service website (ghsghana.org); Ghana Ministry of Health website (moh.gov.gh); WHO Ghana Country Office; World Bank Open Knowledge Repository (openknowledge.worldbank.org); USAID Development Experience Clearinghouse (dec.usaid.gov); African Development Bank repository; OECD iLibrary; Global Fund knowledge repository

**Date searched:** July 2025

**Coverage period searched:** 2000 to 2026

**Method:** Each source was searched manually using site-specific search functions and index browsing. Where site search functionality was limited, Google site-restricted search was applied using the operator site:[domain]. The following query structure was applied across all grey literature sources, adapted to each interface.

**Query applied across grey literature sources:**

("artificial intelligence" OR "AI" OR "digital health" OR "eHealth" OR "health information system" OR "electronic health record") AND ("Ghana" OR "Ghana Health Service") AND ("readiness" OR "implementation" OR "adoption" OR "policy" OR "strategy")

**Supplementary query for broader LMIC grey literature:**

("AI readiness" OR "digital health readiness" OR "health technology adoption") AND ("Sub-Saharan Africa" OR "West Africa" OR "low- and middle-income")

**Document types sought:** Policy documents; national strategies; technical reports; programme evaluations; government white papers; international organisation reports; conference proceedings; working papers

**Language limits:** None

**S1.13 Reference List Checking (Backward Citation Searching)**

**Method:** Manual backward citation checking of all studies included following full-text eligibility assessment. Reference lists of all included studies were screened in full against the eligibility criteria. Additionally, reference lists of key systematic and scoping reviews identified during database searching were screened for potentially eligible primary studies not retrieved through the database searches.

**Date conducted:** July 2025

**Software:** Mendeley Reference Manager used to manage and check references against the existing deduplicated record set

**Language limits:** None

**S1.14 Forward Citation Tracking**

**Interface:** scholar.google.com (cited-by function)

**Date conducted:** July 2025

**Method:** Forward citation tracking was conducted for all included studies and for five pre-identified key papers on AI and digital health in Ghanaian and African health systems used to validate the PubMed strategy. Google Scholar's "Cited by" function was applied to identify subsequent publications citing each key paper. All forward citations retrieved were screened against the eligibility criteria at title and abstract level.

**Key papers tracked:**

Mensah NK, Adzakpah G, Kissi J, Boadu RO, Lasim OU, Oyenike MK, et al. Health professional's readiness and factors associated with telemedicine implementation and use in selected health facilities in Ghana. Heliyon. 2023;9(3):e14501.

Abdulai AF, Adam F. Health providers' readiness for electronic health records adoption: a cross-sectional study of two hospitals in northern Ghana. PLoS ONE. 2020;15(6):e0231569.

Kesse-Tachi A, Asmah AE, Agbozo E. Factors influencing adoption of eHealth technologies in Ghana. Digit Health. 2019;5:2055207619871425.

Antwi-Boasiako AT, Attafuah PYA, Adoquaye E, Nettey VG, Narh CT, Kenu E. Facilitators and barriers in the implementation of a digital surveillance and outbreak response system in Ghana before and during the COVID-19 pandemic. JMIR Form Res. 2023;7:e45715.

Alhassan RK, Kwarteng A, Mensah NK. A scoping review of acceptance and utilization of electronic health records among healthcare professionals in Ghana. Discov Public Health. 2025;22:730.

**Language limits:** None

**S1.15 Journal Hand-Searching**

**Journals searched:** Ghana Medical Journal; Journal of Public Health in Africa; African Health Sciences

**Method:** Manual browsing of tables of contents for the specified coverage period. All article titles and abstracts were screened against the eligibility criteria. Full texts were retrieved for any articles meeting or potentially meeting the eligibility criteria.

**Date conducted:** July 2025

**Coverage period searched:** January 2015 to February 2026 (reflecting the contemporary digital health era and the period of most relevant AI and eHealth development in the Ghanaian context)

**Ghana Medical Journal**

Interface: ghanamedj.org

Years browsed: 2015 to 2025 (all available issues)

Sections of particular relevance: Original articles; review articles; public health and health systems research

**Journal of Public Health in Africa**

Interface: publichealthinafrica.org

Years browsed: 2015 to 2025 (all available issues)

Sections of particular relevance: Original research; systematic reviews; health systems and policy

**African Health Sciences**

Interface: africanhealth sciences.org

Years browsed: 2015 to 2025 (all available issues)

Sections of particular relevance: Health systems; digital health; implementation research

**Language limits:** None

**S1.16 Search Results Summary**

The table below summarises the records retrieved from each source prior to deduplication. Final deduplication and screening counts are documented in the PRISMA 2020 flow diagram.

| **Source** | **Records Retrieved** |
| --- | --- |
| PubMed/MEDLINE | To be reported in final manuscript |
| CINAHL | To be reported in final manuscript |
| Embase | To be reported in final manuscript |
| LILACS | To be reported in final manuscript |
| African Index Medicus | To be reported in final manuscript |
| African Journals Online | To be reported in final manuscript |
| Google Scholar (Standard) | To be reported in final manuscript |
| Google Scholar (Comprehensive) | To be reported in final manuscript |
| CABI Digital Library/SearchRxiv | To be reported in final manuscript |
| WHO IRIS | To be reported in final manuscript |
| ProQuest Dissertations and Theses | To be reported in final manuscript |
| Grey literature | To be reported in final manuscript |
| Reference list checking | To be reported in final manuscript |
| Forward citation tracking | To be reported in final manuscript |
| Journal hand-searching | To be reported in final manuscript |
| **Total pre-deduplication** | **To be reported in final manuscript** |
| **Total post-deduplication** | **To be reported in final manuscript** |

**S1.17 PRESS 2015 Peer Review Checklist Confirmation**

All search strategies documented in this supplementary file were peer reviewed by an independent health information specialist prior to execution, using the PRESS 2015 Evidence-Based Checklist for the peer review of electronic search strategies (McGowan J, Sampson M, Salzwedel DM, Cogo E, Foerster V, Lefebvre C. PRESS Peer Review of Electronic Search Strategies: 2015 guideline statement. J Clin Epidemiol. 2016;75:40-46). The PRESS review confirmed the appropriateness of translation of the review question into search concepts, controlled vocabulary application, text word searching, spelling and syntax, and line combination logic prior to execution of all database searches.
